# Initial orthostatic hypotension in teenagers and young adults

**DOI:** 10.1007/s10286-016-0382-6

**Published:** 2016-09-16

**Authors:** V. K. van Wijnen, M. P. M. Harms, I. K. Go-Schön, B. E. Westerhof, C. T. P. Krediet, J. Stewart, W. Wieling

**Affiliations:** 1 Department of Internal Medicine, University Medical Center Groningen, University of Groningen, Hanzeplein 1, 9713 GZ Groningen, The Netherlands; 2 Department of Internal Medicine, Academic Medical Center, Amsterdam, The Netherlands; 3 Department of Pulmonary Diseases, VU University Medical Center, Amsterdam, The Netherlands; 4 Heart Failure Research Center, Laboratory for Clinical Cardiovascular Physiology, Academic Medical Center, Amsterdam, The Netherlands; 5 Departments of Physiology and Pediatrics, New York Medical College, Valhalla, NY USA; 6 Center for Hypotension, New York Medical College, Hawthorne, NY USA

**Keywords:** Blood pressure, Initial orthostatic hypotension, Syncope, Cardiac output, Vascular resistance, Finapres

## Abstract

**Objective:**

To assess: (1) the frequency of an abnormally large fall in blood pressure (BP) upon standing from supine in patients with initial orthostatic hypotension (IOH); (2) the underlying hemodynamic mechanisms of this fall in BP upon standing from supine and from squatting.

**Methods:**

In a retrospective study of 371 patients (≤30 years) visiting the syncope unit, the hemodynamic response to standing and squatting were studied in 26 patients who were diagnosed clinically with IOH, based on history taking only. In six patients changes in cardiac output (CO) and systemic vascular resistance (SVR) were determined, and the underlying hemodynamics were analyzed.

**Results:**

15/26 (58 %) patients with IOH had an abnormally large initial fall in systolic BP (≥40 mmHg). There was a large scatter in CO and SVR response after arising from supine [ΔCO at BP nadir median −8 % (range −37, +27 %); ΔSVR at BP nadir median −31 % (range −46, +10 %)]. The hemodynamic response after squatting showed a more consistent pattern, with a fall in SVR in all six patients [ΔCO at BP nadir median +23 % (range −12, +31 %); ΔSVR at BP nadir median −42 %, (range −52, −35 %)].

**Interpretation:**

The clinical diagnosis of IOH is based on history taking, as an abnormally large fall in systolic BP can only be documented in 58 %. For IOH upon standing after supine rest, the hemodynamic mechanism can be either a large fall in CO or in SVR. For IOH upon arising from squatting a large fall in SVR is a consistent finding.

## Introduction

Occasional light-headedness and seeing black spots upon standing from supine or arising from a squatting position are experienced in almost all, otherwise healthy, teenagers and young adults [1–4]. In the general population frequent complaints of light-headedness or even (near) syncope are reported to occur in about 20 % of young subjects [1, 5, 6]. Such orthostatic complaints immediately after standing up result from cerebral hypoperfusion due to transient fall in systemic blood pressure (BP) [1].

However, it has not been studied how often a large initial fall in BP can be documented in subjects referred with severe complaints of light-headedness and occasionally (near) syncope during formal cardiovascular reflex testing, i.e., the test characteristics of the active standing test for the diagnosis of initial orthostatic hypotension (IOH) are unknown [1].

In the laboratory both active standing from supine and arising from squatting are used as a test to assess IOH [1, 7]. The initial hemodynamic mechanisms underlying the BP fall upon standing and squatting are thought to be similar [1, 8], but this has never been studied.

In the present study, we report about our experience of more than 10 years with IOH in teenagers and young adults i.e., age ≤30 years. In a retrospective study we analyzed

- The frequency of an abnormally large fall in systolic BP upon active standing from supine in patients with a typical history of severe complaints of IOH occasionally resulting in syncope.
- The hemodynamic mechanism underlying the abnormal large initial fall in BP upon active standing from supine.
- The underlying hemodynamic mechanisms of this initial fall in BP upon standing from supine and from squatting.
- The effectiveness of buttock clenching on IOH using squatting as a provocation.

## Methods

### Study population and measurement protocol (Fig. 1)

From January 2000 up to August 2011, 1651 patients were referred to the syncope unit in the Academic Medical Centre of the University of Amsterdam for the evaluation of transient loss of consciousness or severe presyncope. Out of the 1651 patients, 371 were aged ≤30 years. A primary clinical diagnosis of IOH was present in 26 of these 371 patients (19 males, 7 females) (7 %).

**Fig. 1:**
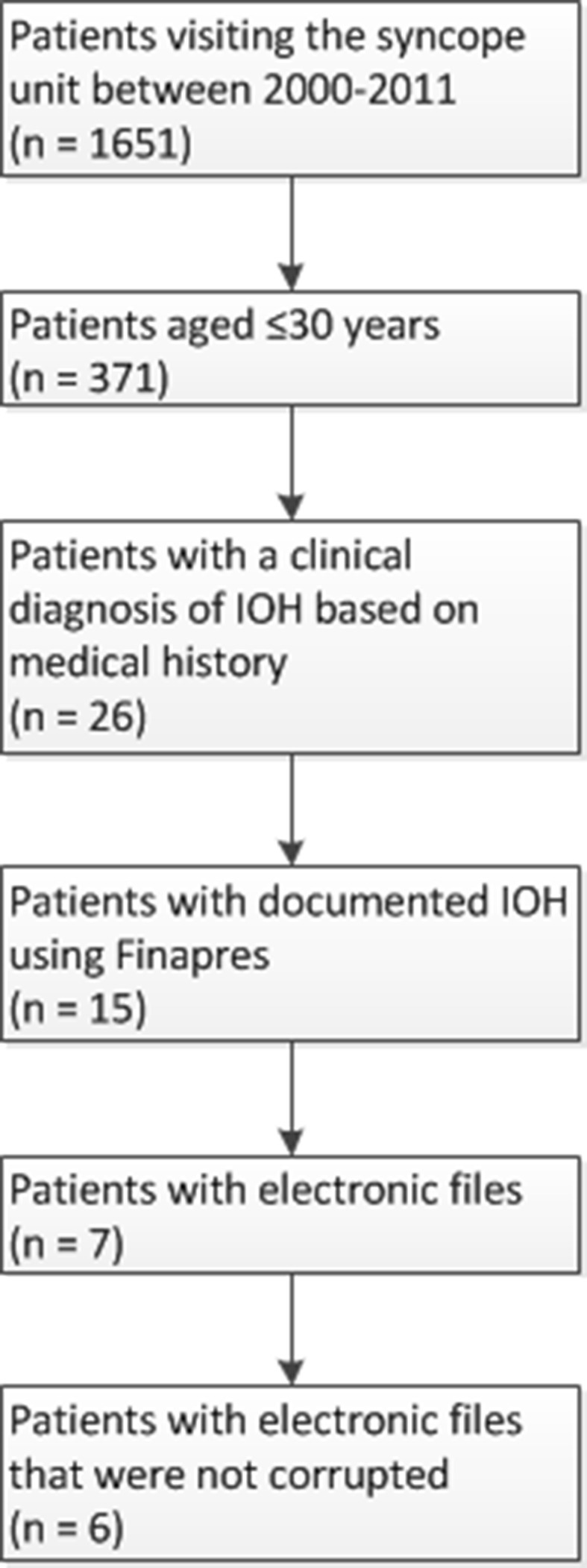
Flowdiagram of patient enrollment

The current analysis focuses on the 26, otherwise healthy, subjects with a primary clinical diagnosis of IOH. These IOH patients had no relevant co-morbidities and did not use vasoactive medications. They all had a history of frequent complaints of severe light-headedness and on occasion syncope in the first 15 s after active standing up.

After consulting the medical ethical committee, an approval was not needed for this retrospective study.

The test protocol used was identical in all 26 retrospective studies. In order to measure the initial orthostatic BP drop and to test for orthostatic hypotension, the active lying-to-standing test was performed with continuous non-invasive BP monitoring. IOH was defined as a transient decrease in systolic BP of >40 and/or >20 mmHg in diastolic BP within the first 15 s of standing [2].

The measurements were performed between 8.00 a.m. and 1.00 p.m. in a temperature controlled room (23 °C). Patients rested supine on a bed for 5–10 min. An active standing-up test was then performed. After a subsequent ~5 min of standing, the patient was instructed to squat for ~1 min and then rise within 1 s and stand for ~30 s. Immediately after standing up from supine and squatting position, the patient was asked whether he had experienced symptoms, e.g., light-headedness.

At the end of the protocol, patients practiced buttock clenching as a physical counterpressure maneuver while arising from the squatting position [9, 10]. In our experience, squatting is especially feasible for patient education, because only 1–2 min of squatting are needed as a provocation compared with at least 5 min of supine rest before active standing. In addition, a squat test has a very good intra-patient reproducibility [9, 10]. The changes in BP were demonstrated to the patient by showing the finger BP tracing on a computer video screen. This biofeedback reveals to patients the relation between symptoms and actual BP values and the effectiveness of the buttock clenching maneuver. Heart rate (HR) and finger arterial pressure were printed by a thermo-paper writer (Thermal array recorder WR 7700, Graphtec, Solingen, Germany).

### Data acquisition and analysis

Non-invasive beat-to-beat BP was measured at the finger with a Finapres Blood Pressure Monitor (TNO-TPD Biomedical Instrumentation, Amsterdam, The Netherlands) or a Nexfin device (BMEYE, Amsterdam, The Netherlands). To avoid hydrostatic pressure differences the hand was held at heart level. From both devices, the brachial artery pressure values reconstructed from finger BP were used.

For a hemodynamic analysis the measured signal was analogue to digital converted at 200 Hz, and stored on a hard-disk for off-line analysis. Mean arterial pressure (MAP) was calculated from the integral of the arterial pressure wave over one beat divided by the corresponding beat interval. HR was computed as the inverse of the inter-beat interval and expressed as beats per min. Beat-to-beat left ventricular stroke volume (SV) expressed in ml was estimated by modeling flow from the arterial pressure waveform (Modelflow, TNO Biomedical Instrumentation) [11]. Cardiac output (CO), expressed in L/min, was the product of estimated SV and HR. Total systemic vascular resistance (SVR), expressed in mmHg s/ml, further called medical units or MU was computed by MAP at heart level divided by the computed CO. Central venous pressure is assumed to be 0 mmHg in this computation. The error involved is negligible. A detailed description about Modelflow can be found in previous literature [11].

### Test periods

1. Baseline: average systolic BP value in the last 10 s in the supine or squatting position before standing up.
2. Lowest point: systolic BP value at the nadir within the first 15 s of standing.
3. Hemodynamics: underlying hemodynamics at the lowest point and recovery were analyzed by computing 5 s averages of SV, CO and SVR. Changes in SV, CO and SVR are given as percentage changes from supine control, respectively, squatting position.

### Statistical analysis

Results are presented as median and range. The effects of standing up from supine and arising from squatting on hemodynamic changes were tested by Wilcoxon sign test. A *p* value less than 0.05 was considered to indicate a statistically significant difference. Actual *p* values are given.

## Results

### Clinical characteristics

Twenty-six patients with IOH as a primary clinical diagnosis (19 males, 7 females) aged ≤30 years were studied. In 15/26 (58 %) of the patients, an abnormally large initial decrease in systolic BP was observed. An abnormally large decrease in diastolic BP (>20 mmHg) was observed in 12 of these 15 patients. In the 11 patients without an abnormally large decrease in systolic BP (>40 mmHg), three subjects had an abnormally large decrease in diastolic BP, matching the definition of IOH. The 15 patients with an abnormally large initial decrease in systolic BP were selected for further analysis (see Discussion).

The median systolic BP fall was −50 mmHg (range −41, −80 mmHg). Electronic files for computer analysis of the underlying hemodynamics were available in 7/15 patients. The missing *N* = 8 were older tracings on paper, but with no electronic files available. One electronic file was unsuitable for hemodynamic analysis due to hardly no pulse pressure during the hypotensive period induced by arising from the squatting position. Thus 6 files [3 males, 3 females; age 21 (range 14–29) years; BMI 20 (range 18–22) kg/m^2^; weight 64 (range 55–75) kg] were available for analysis (Table 1). A fall in systolic BP ≥20 mmHg was not observed during the 5-min standing period. Thus conventional orthostatic hypotension was not observed.

**Table 1:** Clinical characteristics of six patients with the clinical diagnosis of initial orthostatic hypotension and an abnormally large initial fall in blood pressure of whom electronic recordings were available

Patients	
Gender	3 male; 3 female
Age	21 (14–29) years
Height	179 (169–186) cm
Weight	64 (55–75) kg
BMI	20.2 ± 1.9 kg/m^2^
Baseline upper arm cuff pressure	120 (104–142)/69 (59–88) mmHg
Baseline continuous pressure	121 (±14)/66 (±13) mmHg
Baseline heart rate	76 (±15) bpm

The clinical history of these six IOH patients consisted of severe episodes of light-headedness upon standing, with occasionally seeing black spots. Syncope, shortly after standing up, was reported in four patients. In all of them, the episodes of light-headedness occurred after standing up and walking about five steps. In addition to severe complaints of IOH, episodes of vasovagal fainting were reported by 3/6 patients. None of the patients complained about episodes of (near) fainting on arising from squatting.

### BP response to active standing from supine and from squatting (Fig. 2)

The initial fall in systolic BP during standing up from supine amounted to a median of −52 mmHg (range −46, −66 mmHg). The median time at the nadir was 8.5 s (range 6.6–10.0 s). Within 20 s after standing up from supine, systolic BP had recovered to values around supine control values, with an overshoot in 4/6 patients (patients 2, 3, 5, 6). Five out of six patients (patients 2–6) reported light-headedness 5–10 s after the onset of standing up.

**Fig. 2:**
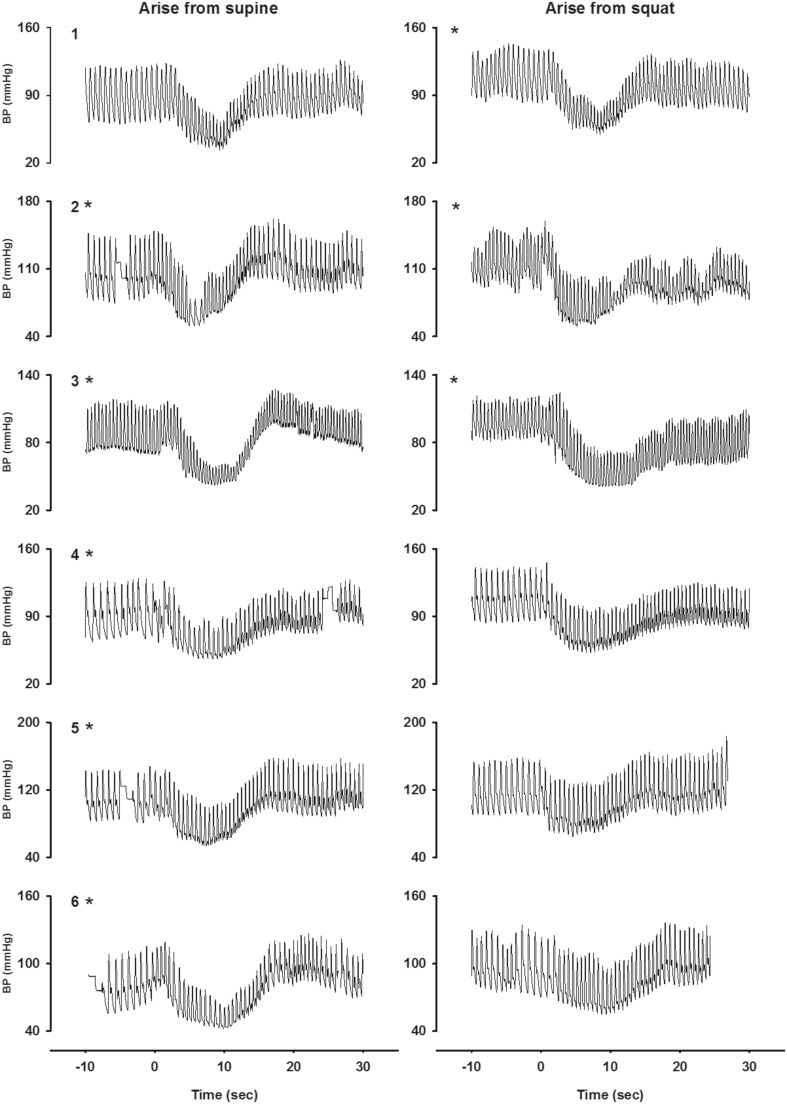
Initial BP responses upon arising from supine and squatting. The individual beat-to-beat systolic BP responses are given between the last 10 s of supine rest and squatting position until the first 20–30 s after arising. *Asterisk* patients with symptoms of light-headedness 5–10 s after arising. *BP* blood pressure

The initial fall in systolic BP during arising from squatting amounted to a median of −49 mmHg (range −32, −70 mmHg) (*p* = 0.13 vs. standing up). The median at the nadir was 8.5 s (range 7.7–9.5 s) (*p* = 0.41 vs. standing up). Within 20 s after standing up from squatting, systolic BP had not recovered to values around supine control values in 4/6 patients (patients 1–4). Patients 1–3 reported light-headedness 5–10 s after arising standing up from squatting.

In some subjects, a large initial fall in BP was present with both maneuvers (patients 1, 3), whereas in other patients only a small fall in systolic BP was present upon active standing from the squatting position (patients 5, 6). The peak HR rise at the moment of the nadir upon active standing from supine amounted to 34 bpm (range 26–42 bpm). The peak HR rise at the moment of the nadir upon standing from squat amounted to 28 bpm (range 8–46 bpm).

### Hemodynamic response to active standing from supine and from squatting (Fig. 3)

The CO and SVR responses at the moment of the BP nadir during active standing from supine showed a large scatter. Both, a large fall in CO and/or a fall in SVR, could underlie this large initial BP dip. At the moment of the nadir, the change in CO amounted to −8 % (range −37, +27 %) (*p* = 0.56) and in SVR to −31 % (range −46, +10 %) (*p* = 0.16).

**Fig. 3:**
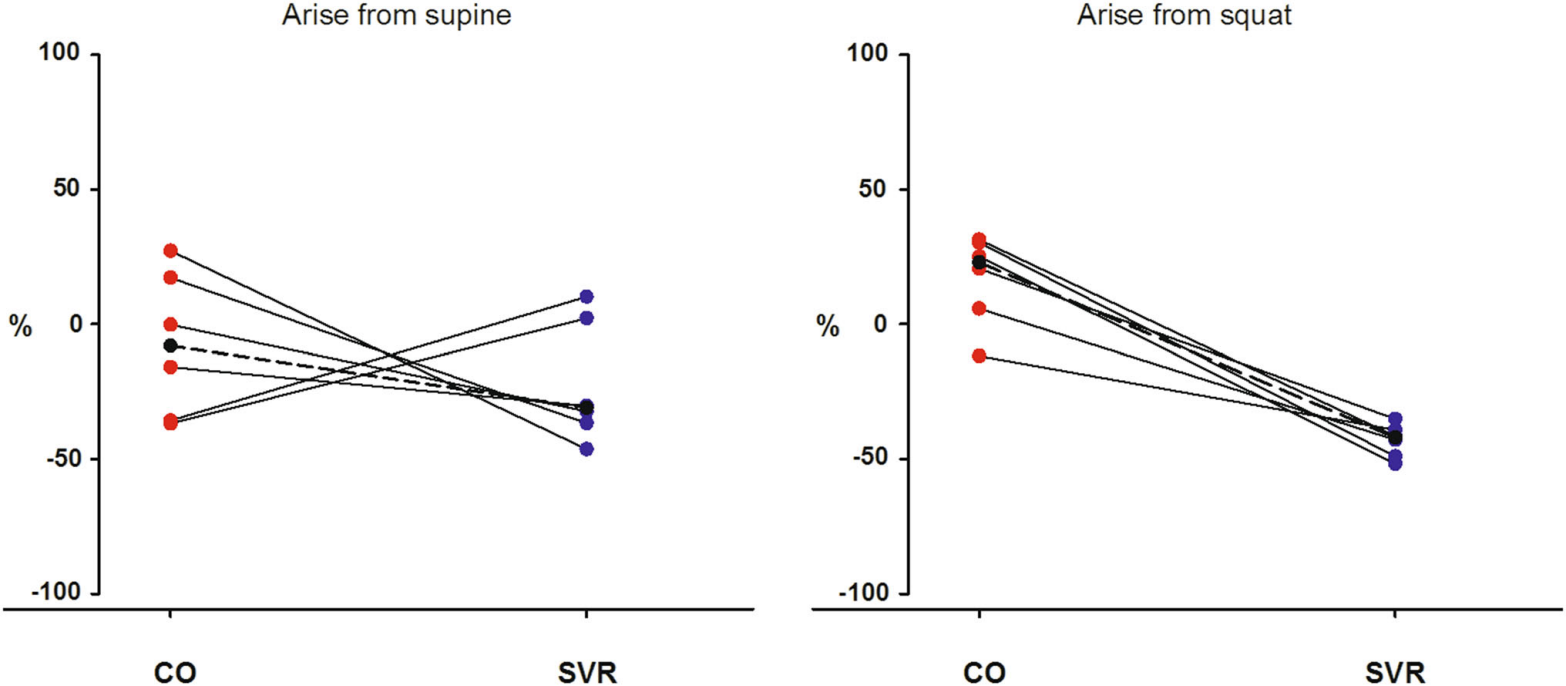
Hemodynamic responses underlying the initial BP dip during active standing from supine (**a**) and from squatting (**b**). The CO and SVR responses are estimated by modeling flow from the arterial pressure waveform. SVR and CO are connected and given in percentages of change (from supine control or squat to the initial BP dip). *BP* blood pressure, *CO* cardiac output, *SVR* systemic vascular resistance

The hemodynamics underlying arising from the squatting position showed a much more consistent pattern. In the squatting position SVR fell in all six patients to −42 % (range −52, −35 %) after standing up (*p* = 0.03). At the moment of the nadir, the change in CO amounted to +23 % (range −12, +31 %) (*p* = 0.09) The median changes in CO between supine to standing (−8 %) and squat to stand (+23 %) differed (*p* = 0.03). In SVR these changes from supine to standing (−31 %) and squat to stand (−42 %) did not differ (*p* = 0.06). However, the starting value of the SVR in the squatting position was higher than in the supine position (*p* = 0.03) while CO was comparable (*p* = 0.22).

### Effects of buttock clenching (Fig. 4)

The effect of buttock clenching on IOH was assessed at the end of the protocol by arising from squatting with and without buttock clenching, demonstrating the differences to the patients on the computer screen. In all six IOH patients, buttock clenching was effective in ameliorating the initial fall in BP. In patients 1–3 with a large transient fall in BP and symptoms of light-headedness the abnormally fall in systolic BP decreased by 15–20 mmHg and symptoms were no longer present. In patient 3, the most symptomatic patient, the effect of buttock clenching was also examined after arising from supine. This patient experienced severe complaints during active standing without skeletal muscle tensing. By applying moderate buttock clenching, the fall in BP and pulse pressure was much less pronounced and the symptoms were minimal. The BP increased markedly after standing up, by applying maximal whole body tensing.

**Fig. 4:**
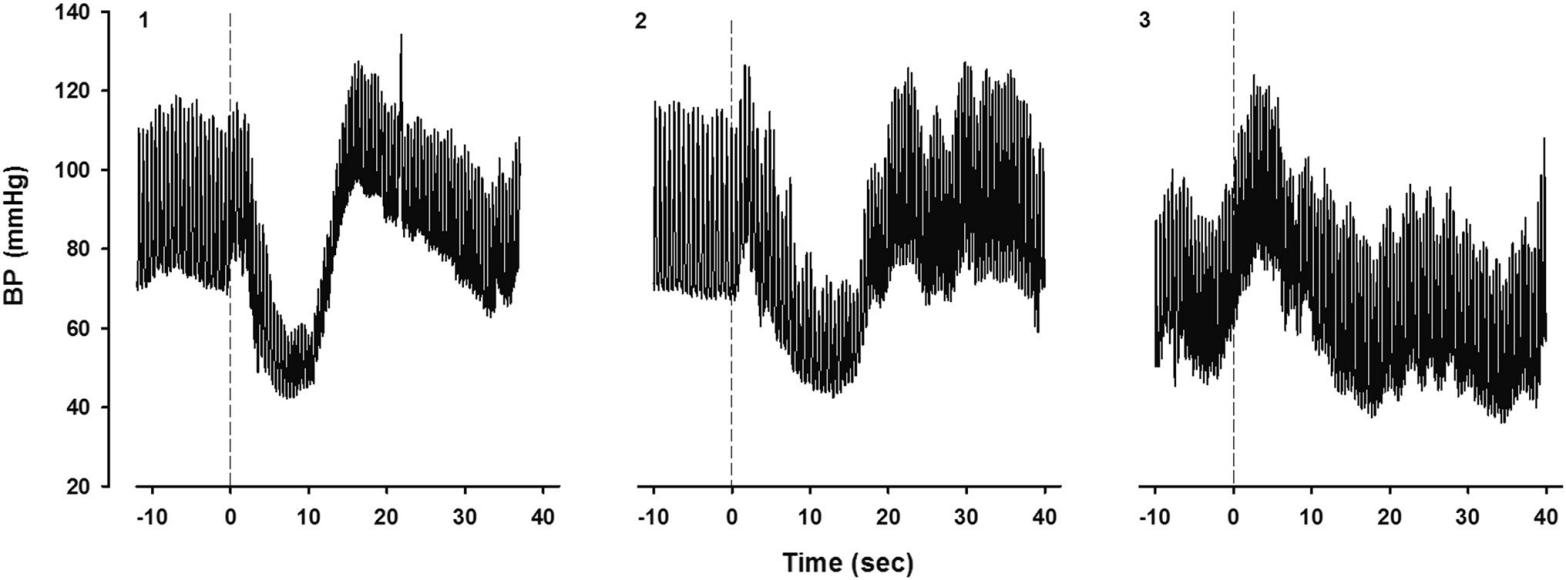
Effect of buttock clenching in patient 3. Shown is the continuous BP during the last 10 s in supine position and the first 40 s while standing without muscle tension (**a**), moderate muscle tension (**b**) and maximal muscle tension (**c**). *BP* blood pressure

## Discussion

Initial orthostatic hypotension is the most common form of orthostatic intolerance in the young [1, 8, 12].

The main findings of the present study are:

1. A clinical history of IOH was a frequent reason for referral to our syncope unit in patients ≤30 years. It occurred in 26/371 (7 %) of the referred patients. An abnormally large fall in systolic BP (≥40 mmHg) was present in only 15/26 (58 %) of these IOH patients.
2. The physiological mechanisms underlying IOH after standing up from supine vary. Both, a large fall in CO and in SVR may occur, whereas after arising from the squatting position a fall in SVR is a consistent underlying mechanism.
3. Effect of buttock clenching is evident. The BP increases markedly by applying this maneuvre.

### The role of the medical history and BP measurement in diagnosing IOH

Current views on diagnostic testing [13] are of direct relevance to the question whether a typical clinical history is sufficient to diagnose IOH or whether the documentation of an abnormally large transient fall in systolic BP is needed in addition.

The therapeutic aim in young patients with severe symptomatic IOH is functional recovery and improvement of quality of life. If the complaints of transient light-headedness, seeing black spots or even (near-) syncope occur upon active standing, there is hardly a differential diagnosis. Other conditions with “dizziness” and apparent loss of consciousness that may be elicited by standing up, like benign paroxysmal positional vertigo, anxiety, psychogenic pseudosyncope and malingering, have a different presentation. The latter two conditions occur upon active standing, but are in our experience extremely rare (<1/1000 presentations).

**Fig. 5:**
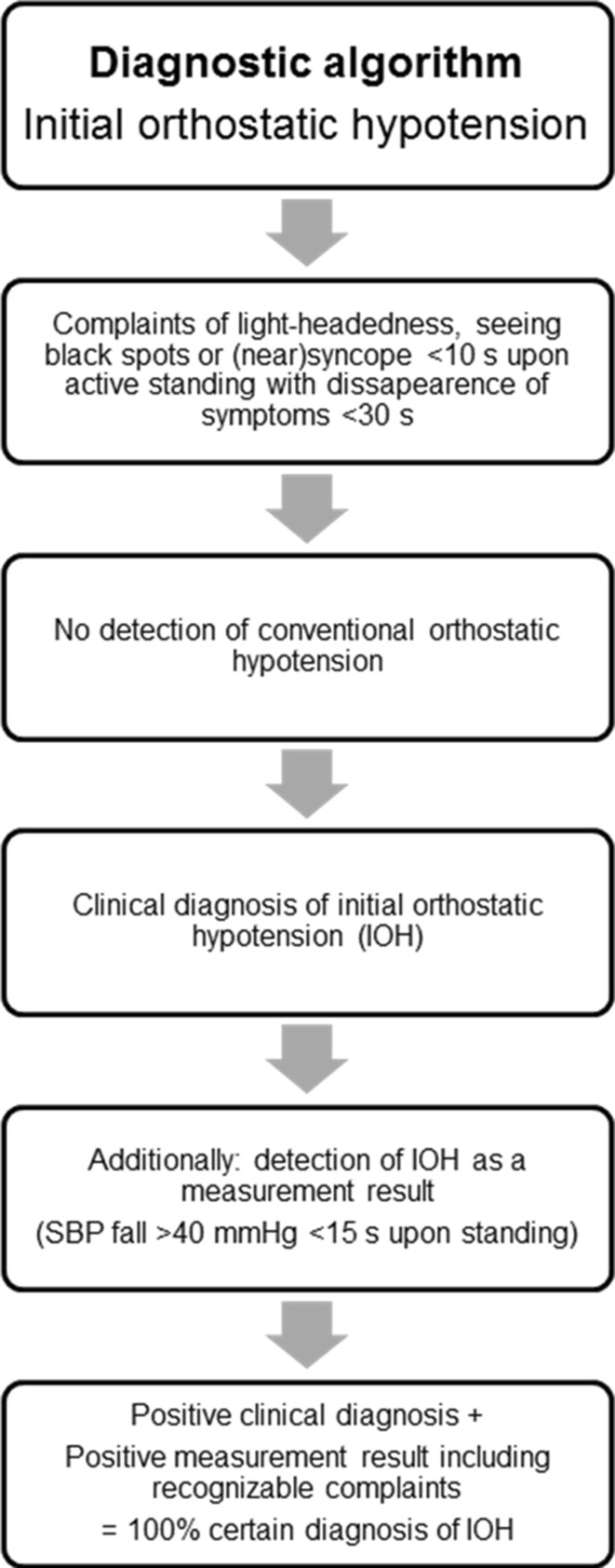
Diagnostic algorithm of initial orthostatic hypotension. A typical clinical history is sufficient to diagnose initial orthostatic hypotension, if there is an absence of conventional orthostatic hypotension (systolic BP ≥20 mmHg and/or diastolic BP ≥10 mmHg <3 min of standing). The diagnosis becomes 100 % certain if an abnormally large fall in systolic BP (>40 mmHg) is documented <15 s of standing, accompanied with typical symptoms. *BP* blood pressure

Considering the lack of malignant causes of syncope for complaints of IOH, we suggest that a typical clinical history of IOH alone suffices to reach to a highly likely (80–100 % certain) diagnosis. In case an abnormal fall in systolic BP (>40 mmHg) is documented in the laboratory accompanied by typical complaints, the diagnosis becomes 100 % certain (Fig. 5). Twelve out of the 15 patients with an abnormally large initial decrease in systolic BP also had an abnormally large decrease in diastolic BP. Only 3/26 patients had an isolated abnormally large diastolic decrease in BP. Overall, the clinical information of the systolic and diastolic BP changes matched in 20/26 patients. We used a systolic BP decrease of >40 mmHg in this study. This is in accordance with a study with community-dwelling elderly that documented that only a fall in systolic BP was associated with symptoms of orthostatic intolerance [14]. In healthy teenagers and young adults this has not been studied.

Four patients with IOH and syncope reported sudden black out and fainting after standing up and walking some five steps. This presentation of symptoms, occurring after walking a few steps, is typical in our experience. However, it has never been studied whether walking a few steps after standing up increases the initial BP fall compared to standing still after arising. The interval between the moment of standing up and the onset of syncope corresponds to the latency between the onset of cerebral hypoperfusion and symptoms, which is approximately 6 s [15]. Cerebral autoregulation is too slow to make rapid adjustment and additionally the BP drop is outside the range of cerebral autoregulation [16].

### Physiological mechanisms underlying the initial BP fall after standing up from supine

Previous studies in healthy teenagers and young adult subjects using beat-to-beat measurement of SV with calculation of CO and SVR, have established that CO actually increases with the onset of standing up, whereas SVR falls markedly [17–20]. More invasive measurements have identified immediate elevation in right atrial pressure (RAP) upon standing from supine, similar to what happens at the onset of upright exercise [20]. These observations support the hypothesis that a central shift in blood volume, due to leg and abdominal vascular compression, occurs at the onset of whole body exercise, increasing cardiac filling and CO [8].

The initial fall in BP upon standing is due to a mismatch of this increase in CO and a decrease in SVR and does not occur, or is far less pronounced, on a passive change of posture [12, 17]. Three factors have been brought forward to explain the large fall in SVR [1, 8]:

- Activation of cardiopulmonary baroreceptors by the elevation in RAP at the onset of whole body exercise and subsequent sympathetic withdrawal.
- Rapid vasodilation in the contracting leg muscles with the effort of standing.
- An increase in the A–V pressure gradient (mechanical effect).

The first factor is unlikely as the time constant for vascular relaxation is too long. In addition, it has been shown that reflex vasodilation in the forearm was not observed during arising from the squatting position [8]. The second and third factors have been both documented to be involved [8, 12]. After prolonged supine rest the effects of the A–V gradient are prominent [12].

### Physiological difference in mechanisms underlying IOH after standing and squatting

The initial hemodynamic mechanisms underlying the BP fall upon standing are thought to be similar after supine rest and squatting [8]. This study documents that this view is not correct, the hemodynamic mechanisms underlying IOH arising from standing and squatting in patients with IOH are different.

This study confirms that the fall in BP after arising from squatting is based primarily on a fall in SVR [1, 8, 21]. In accordance with previous studies in healthy young adults [9], the CO at the moment of the nadir was increased (+23 %). However, for the large initial fall in BP upon standing after supine rest, the hemodynamic mechanism can be either a large fall in CO or in SVR (Fig. 3). CO at the moment of the nadir after standing from supine was, in contrast to arising from squatting, tended to be lower (−8 %).

The rapid dilatation in leg muscles upon arising from a squatting position can be attributed to the combination of relative ischemia due to compression of blood vessels, active muscle contraction and elevated lower limb arterio-venous pressure gradient [1, 8, 12, 21].

The initial fall in BP after standing from supine and squatting was similar in our study (−52 mmHg vs. −49 mmHg). Earlier studies have shown that the trough of BP after arising from squat is deeper than the trough after standing up from a supine position [1]. The different finding in our study can be explained by the short duration of squatting [8].

### Effect of buttock clenching

In accordance with previous studies, buttock clenching was very effective to abort IOH (Fig. 4) [9, 10, 22]. The effect of buttock clenching on complaints during long-term follow-up can thereby be used as the reference that the diagnosis of IOH is correct [23–25]. This emphasizes an important aspect of laboratory testing, namely the effects of physical counterpressure maneuvers can be demonstrated to the patient on a video screen [22]. An additional advantage to use squatting for instructions to patients is that it can be demonstrated that squatting itself increases BP instantaneously and thereby can be used as an emergency measure to abort an impending syncope [9–11].

### Limitations

This study has several limitations. The first limitation concerns the small number of study subjects, this as a result of the criteria used for this study. The second limitation is the retrospective nature of this study. As a result, not all suitable files could be used. Also more specific patient details concerning history, complaints and other measurements were not available. Nevertheless, with an experience of more than 10 years of IOH and 1651 syncope patients visiting the syncope unit, this study answers the objectives and puts a new light on the current understanding of the hemodynamics underlying the large initial fall in systolic BP after standing from supine and squatting position. The present study can be considered as a starting point to discuss the role of standing from supine and arising from squatting to diagnose, study and manage the mechanism of IOH.

## Conclusion

IOH is a frequent cause of syncope and the clinical diagnosis is based on history taking. An abnormally large fall in systolic BP can only be documented in laboratory settings in 58 % of the subjects. For IOH upon standing after supine rest, the hemodynamic mechanism can be either a large fall in CO or in SVR, for IOH upon arising from squatting a large fall in SVR is a consistent finding.

## Abbreviations

BP: Blood pressure
CO: Cardiac output
HR: Heart rate
IOH: Initial orthostatic hypotension
MAP: Mean arterial pressure
RAP: Right atrial pressure
SV: Stroke volume
SVR: Systemic vascular resistance
